# Activation of factor VII-activating protease in human inflammation: a sensor for cell death

**DOI:** 10.1186/cc10131

**Published:** 2011-04-05

**Authors:** Femke Stephan, Jan A Hazelzet, Ingrid Bulder, Marja A Boermeester, JW Olivier van Till, Tom van der Poll, Walter A Wuillemin, Lucien A Aarden, Sacha Zeerleder

**Affiliations:** 1 Department of Immunopathology, Sanquin Research at CLB and Landsteiner Laboratory of the AMC, Plesmanlaan 125, 1066 CX Amsterdam, The Netherlands; 2 Department of Pediatrics, Division of Pediatric Intensive Care, Erasmus MC - Sophia Children's Hospital, Dr Molewaterplein 60, 3015 GJ Rotterdam, The Netherlands; 3 Department of Surgery, Academic Medical Center, University of Amsterdam, Meibergdreef 9, 1105 AZ Amsterdam, The Netherlands; 4 Center for Infection and Immunity Amsterdam (CINIMA) and Center for Experimental and Molecular Medicine (CEMM), Academic Medical Center, University of Amsterdam, Meibergdreef 9, 1105 AZ Amsterdam, The Netherlands; 5 Division of Hematology and Central Laboratory of Hematology, Luzerner Kantonsspital Luzern, CH-6000 Lucerne 16, Switzerland; 6 Department of Hematology, Academic Medical Center, University of Amsterdam, Meibergdreef 9, 1105 AZ Amsterdam, The Netherlands

## Abstract

**Introduction:**

Cell death is a central event in the pathogenesis of sepsis and is reflected by circulating nucleosomes. Circulating nucleosomes were suggested to play an important role in inflammation and were demonstrated to correlate with severity and outcome in sepsis patients. We recently showed that plasma can release nucleosomes from late apoptotic cells. Factor VII-activating protease (FSAP) was identified to be the plasma serine protease responsible for nucleosome release. The aim of this study was to investigate FSAP activation in patients suffering from various inflammatory diseases of increasing severity.

**Methods:**

We developed ELISAs to measure FSAP-C1-inhibitor and FSAP-α_2_-antiplasmin complexes in plasma. FSAP-inhibitor complexes were measured in the plasma of 20 adult patients undergoing transhiatal esophagectomy, 32 adult patients suffering from severe sepsis and 8 from septic shock and 38 children suffering from meningococcal sepsis.

**Results:**

We demonstrate plasma FSAP to be activated upon contact with apoptotic and necrotic cells by an assay detecting complexes between FSAP and its target serpins α_2_-antiplasmin and C1-inhibitor, respectively. By means of that assay we demonstrate FSAP activation in post-surgery patients, patients suffering from severe sepsis, septic shock and meningococcal sepsis. Levels of FSAP-inhibitor complexes correlate with nucleosome levels and correlate with severity and mortality in these patients.

**Conclusions:**

These results suggest FSAP activation to be a sensor for cell death in the circulation and that FSAP activation in sepsis might be involved in nucleosome release, thereby contributing to lethality.

## Introduction

Sepsis is characterized by an extensive inflammatory response including cytokine generation, activation of plasmatic cascade systems and inflammatory cells leading to organ dysfunction and in many cases to death [1]. Extensive cell death as a downstream effect of these mediators was postulated to be critically involved in the development of organ dysfunction [2]. Indeed, several studies in animal models for sepsis and in sepsis patients demonstrated widespread apoptosis of lymphoid tissue and to a lesser extent of parenchymal cells [3-5]. As a result of extensive cell death circulating nucleosomes could be measured in sepsis patients [6]. Moreover, nucleosomes could be detected in patients with severe peritonitis [7]. Levels of circulating nucleosomes and pulmonary nucleosome levels were demonstrated to correlate with severity and outcome in sepsis patients [6,7]. Recent findings suggest that these circulating nucleosomes play a crucial role in inflammation. Circulating histones 3 and 4 turned out to be highly cytotoxic and to mediate lethal effects in sepsis [8]. We recently showed that Factor VII-activating protease (FSAP) in plasma can remove nucleosomes from late apoptotic cells [9,10].

FSAP, also known as plasma hyaluronic acid binding protein 2 (HABP2), is a serine protease which circulates in plasma as an inactive single-chain molecule of 64 kDa. It is proteolytically converted in its active two-chain form consisting of a 50 kDa heavy and a 28 kDa light chain connected by a disulfide bond [11]. Purified plasma-derived FSAP is described to be susceptible to autoactivation [12]. Recently published data suggest that purified FSAP can bind and be activated by negatively charged polyanions such as heparin, polyphosphates, RNA and DNA [11,13-15]. In purified systems, various serine protease inhibitors (serpins) such as C1-inhibitor (C1-inh), α_2_-antiplasmin (AP), antithrombin III (AT-III) and plasminogen activator inhibitor-1 (PAI-1) [11,16-19] were reported to inhibit the amidolytic activity of plasma-derived activated FSAP. In plasma, C1-inh has been reported to be the main inhibitor of activated FSAP [16].

Since compounds of circulating nucleosomes induce lethality in sepsis [8], we suggest FSAP activation in sepsis to be involved in nucleosome release. The aim of this study is to investigate FSAP activation in patients suffering from inflammatory diseases of increasing severity. Due to a lack of specific substrate and its susceptibility for autoactivation measurement of FSAP activation is troublesome. Therefore, we set up assays to follow FSAP activation in plasma. We made use of the fact that upon activation FSAP quickly forms stable covalent complexes with its plasma inhibitors. We set up ELISAs to measure FSAP-serpin complexes. By means of these assays we measured FSAP activation in patients after surgery, patients with severe sepsis, septic shock and meningococcal sepsis.

## Materials and methods

### Patients

The study was approved by the institutional medical ethics committees of the centers involved, and from all study participants or legal representatives written informed consent was obtained.

#### Healthy controls

Citrated plasma was collected from 20 healthy Dutch lab workers.

#### Post-operative acute-phase response

Twenty consecutive patients with resectable adenocarcinoma of the middle or distal esophagus or esophagogastric junction were studied. Pre-operative and peroperative investigations revealed no distant metastases, and none of the patients received (neo-) adjuvant chemotherapy or radiotherapy [20]. EDTA and citrated blood was sampled pre-operatively (Day 0) and on days 1, 3, 5, 7, and 10 after surgery and the blood samples were stored at -80°C until analysis.

#### Severe sepsis and septic shock

Patients of the medical and surgical ICU were eligible if they met the inclusion criteria for severe sepsis and septic shock according to the definitions of the American College of Chest Physisians (ACCP) consensus conference [21]. Patients were followed for 90 days or until death. The sepsis patients participated in a randomized, double-blind placebo controlled pilot study to study the efficacy of C1-inhibitor in sepsis [22]. EDTA and citrated blood was sampled at inclusion into the study before the administration of C1-inhbitor or placebo, respectively. The blood samples were stored at -80°C until analysis. Clinical parameters, organ dysfunction scores and acute phase parameters were assessed as recently described [6].

#### Meningococcal sepsis

Children between 1 month and 18 years of age with septic shock and petechiae/purpura were enrolled in this study. The children were included in a randomized, double-blind placebo controlled dose-finding study to test the efficacy of plasma-derived protein C (PC) in sepsis. Twenty-eight received PC in escalating doses, whereas 10 received a placebo. Arterial citrated and EDTA blood samples were collected within two hours after admission (before the start of PC or the placebo) and several time points afterwards and stored at -80°C until analysis. The clinical characteristics of these patients are described in detail elsewhere [23]. Clinical parameters, organ dysfunction scores and acute phase parameters were assessed as recently described [23].

### Reagents

Mouse monoclonal antibodies to FSAP (anti-FSAP-4 and anti-FSAP-9), to complexed C1-inhibitor (KOK-12), to α_2_-antiplasmin (AAP-20), and a control antibody (anti-IL6) were prepared at our department (all IgG1κ) [10,24]. PE-labeled rabbit-anti-mouse F(ab')2 antibody was obtained from Dako (Glostrup, Denmark). Iscove's modified Dulbecco's medium was obtained from BioWhittaker Europe (Verviers, Belgium). Fetal calf serum was obtained from Bodinco BV (Alkmaar, The Netherlands). Penicillin and streptomycin were obtained from Gibco/Invitrogen (Groningen, The Netherlands). Etoposide, ß-mercaptoethanol, RNase, and nitro blue tetrazolium and 5'-bromo-4'-chloro-3'-indolyl phosphate (NBT/BCIP) were obtained from Sigma (Zwijndrecht, The Netherlands). NuPage 4 to 12% polyacrylamide gels, sample buffer, dithiothreitol (DTT) and nitrocellulose membranes were obtained from Invitrogen (Groningen, The Netherlands). Western blocking reagent was obtained from Roche Diagnostics (Mannheim, Germany). Streptavidin-alkaline phosphatase was obtained from Mabtech (Nacka Strand, Sweden). High performance ELISA buffer (HPE) and Poly-HRP-labeled streptavidin were obtained from Sanquin (Amsterdam, The Netherlands). (3,5,3',5')-tetramethylbenzidine (TMB) was obtained from Merck (Darmstadt, Germany). CNBr-activated sepharose and Protein G Sepharose was obtained from Pharmacia Biochem (Uppsala, Sweden).

### Cell culture and induction of apoptosis and necrosis

Jurkat cells were cultured in culture medium (Iscove's Modified Dulbecco's Medium (IMDM) containing 5% (v/v) fetal calf serum (FCS), penicillin (100 IU/ml), streptomycin (100 μg/ml) and 50 μM ß-mercaptoethanol. Before apoptosis and necrosis induction, cells were washed three times with culture medium without FCS by centrifugation at 360 × g for 10 minutes and resuspended in culture medium without FCS. Cells (1 × 10^6 ^cells/ml) were incubated for 48 h with etoposide in a final concentration of 200 μM to induce apoptosis. For necrosis induction, cells were incubated with 0.3% H_2_O_2 _at 60°C for 60 minutes.

### Recalcified plasma

In previous experiments recalcified citrated plasma was used instead of serum clotted in the presence of cells since microparticles in serum obscure the FACS analysis [9]. It turned out that FSAP was not activated upon recalcification and that recalcified citrated plasma removed nucleosomes from apoptotic cells as efficiently as serum [9,10]. In the text, recalcified citrated plasma is denoted as r-plasma. Blood was obtained from healthy donors in vials containing a final concentration of 10 mM sodium citrate and were centrifuged two times at 1,300 × g. Citrated plasma was recalcified with 20 mM CaCl_2 _in a glass vial and incubated at 37°C for 30 minutes. Subsequently the plasma was incubated at 4°C for 30 minutes and the formed clot was removed. The r-plasma was stored at -20°C until use. All donors were homozygous for the wild-type form of FSAP [25].

### FSAP activation in recalcified plasma

FSAP was activated as recently described [10]. Apoptotic, necrotic or living Jurkat cells were washed in HN-buffer (10 mM Hepes, 140 mM NaCl, pH 7.2) and resuspended in HN-buffer at a concentration of 2 × 10^6 ^cells/ml. RNase was added to the cells to a final concentration of 10 U/ml and incubated for 30 minutes at 37°C. R-plasma was incubated for 30 minutes at 37°C with living, necrotic or apoptotic Jurkat cells (0.5 × 10^6 ^cells/ml) in HN-buffer.

### Binding of FSAP to cells

Apoptotic or living Jurkat cells were washed in HN-buffer (10 mM Hepes, 140 mM NaCl, pH 7.2) and resuspended in HN-buffer at a concentration of 2 × 10^6 ^cells/ml. RNase was added to the cells to a final concentration of 10 U/ml and incubated for 30 minutes at 37°C. R-plasma was incubated for 30 minutes at 37°C with living, necrotic or apoptotic Jurkat cells (0.5 × 10^6 ^cells/ml) in HN-buffer. After three washes with buffer B-0.5% BSA (10 mM Hepes, 150 mM NaCl, 5 mM KCl, 2 mM CaCl2 and 2 mM MgCl2) cells were incubated with an anti-FSAP antibody or an irrelevant anti-IL6 antibody for 15 minutes. Cells were washed and stained with a PE-labeled rabbit-anti-mouse F(ab')2 antibody. Cells were washed, resuspended in 150 μl buffer B- 0.5% BSA and the samples were analyzed by flow cytometry using BD LSRII flow cytometer (Becton Dickinson, Mountain View, CA, USA). Cells being double positive for propidium iodide and Annexin V were considered to be apoptotic.

### Immunoprecipitation and Western blotting

R-plasma was incubated with anti-FSAP-4 antibody coupled to CNBr-activated sepharose (10 mg mAb to 500 mg of sepharose) for two hours at room temperature (RT) with gentle shaking [10]. The sepharose was washed three times with PBS, 0.02% Tween 20, 500 mM NaCl. Proteins attached to the beads were eluted by heating to 90°C for 10 minutes with SDS-PAGE sample buffer containing 50 mM dithiothreitol (DTT) and samples were applied to NuPage 4 to 12% polyacrylamide gels. After electrophoresis, Western blotting was performed on the gel according to the Western blotting protocol Novex^®^. In brief, samples were blotted onto a nitrocellulose membrane and blocked with 1% Western blocking reagent in TBS-T (10 mM Tris pH 8.0, 150 mM NaCl, 0.1% Tween 20). Subsequently they were incubated with biotinylated anti-FSAP-9 recognizing the light chain of FSAP (1 μg/ml). The membranes were washed and Streptavidin-alkaline phosphatase in TBS-T 0.5% Western blocking reagent (dilution 1:500 v/v) was added. The blotting membranes were developed using NBT/BCIP and the reaction was stopped with distilled water.

### Identification of plasma inhibitors of FSAP

Monoclonal anti-FSAP-4 or the irrelevant anti-IL6 was coupled to CNBr-activated sepharose (10 mg mAb to 500 mg of sepharose). Columns were washed five times with start buffer (10 mM Hepes, 1M NaCl, 0.02% Tween 20, pH 7.2). R-plasma incubated with apoptotic Jurkat cells or r-plasma incubated with buffer was applied to the column at a flow rate of 1 ml/minute followed by washing with five column volumes of start buffer. Elution was performed with 0.1M glycine, 140 mM NaCl, 0.02% Tween 20, pH 2.5. The eluate was adjusted to pH 7 with 1M tris-HCl, pH 8. Subsequently eluates were IgG-depleted. The Protein G Sepharose was washed three times with PBS and 1 ml of eluate was added to 0.5 ml of Protein G Sepharose and incubated head-over-head overnight at 4°C. The supernatants were diluted in SDS-PAGE sample buffer and applied to NuPage 4 to 12% polyacrylamide gels. Gels were stained with Coomassie Blue or silver staining by using a silverstaining kit.

MALDI-TOF peptide mass fingerprinting and MALDI-TOF/TOF peptide sequencing analysis was performed on the excised Coomassie Blue stained protein band at Eurosequence B.V. (Meditech Center, Groningen, The Netherlands). Identification was performed by database search using MASCOT software.

### Determination of FSAP-AP and FSAP-C1-inh complexes by ELISA

For detection of complexes of FSAP with C1-inh and AP, 96-well microtiter plates (maxisorp, Nunc) were coated for four hours at RT with a monoclonal antibody specific for C1-inh in complex [24] or with a monoclonal antibody against AP both at 2 μg/ml diluted in PBS. All further incubations were performed at RT under shaking conditions. After each step, the wells were washed five times with washing fluid (PBS, 0.02% Tween 20) with a Microplate Autowasher (Bio-tek Instruments, Inc. Winooski, VT, USA). All incubation steps were performed in high performance ELISA buffer (HPE). Plasma samples were added to the plate and incubated for 60 minutes at RT. Wells were washed five times and biotinylated anti-FSAP-4 (1 μg/ml) was added and incubated for 60 minutes. After five washes streptavidin-polymerized horseradish peroxidase (poly-HRP) conjugate (dilution 1:10,000 v/v) diluted in HPE was added to each well and incubated for 20 minutes. Plates were developed by an addition of 100 μg/ml 3,3',5,5'-tetramethylbenzidine and 0.003% hydrogen peroxide in 0.11 M sodium acetate buffer pH 5.5. Coloring reaction was stopped by adding 100 μl of 2 M H_2_SO_4_. The absorbance was measured at 450 nm with an ELISA plate reader (Multiskan, Thermo Labsystems, Breda, The Netherlands). As standard curve we used citrated r-plasma incubated with apoptotic cells in presence of 20 mM EDTA as described in detail above. Citrated plasma of 20 healthy donors was incubated with apoptotic cells and FSAP-serpin complexes were measured. The median of the level of complexes in these controls was arbitrarily set as 0.5 AU/ml since plasma was diluted 1:1 (v/v) with apoptotic cells. The intra- and inter assay coefficient of variation of the FSAP-AP complex ELISA was 9% and 8%, respectively. Both, the intra- and inter-assay coefficient of variation of the FSAP-C1-inh complex ELISA was 5%.

### Nucleosome ELISA

Nucleosome levels were determined by an ELISA as recently described [9]. Briefly, monoclonal antibody CLB-ANA/60, which recognizes histone 3 was used as a catching antibody. Biotinylated F(ab')2 fragments of CLB-ANA/58, which recognizes an epitope exposed on complexes of histone 2A, histone 2B and dsDNA, in combination with poly-HRP were used for detection.

### Statistics

Results are expressed as mean ± SEM/median with range. The Mann-Whitney rank-sum test was used to assess differences between groups at a given time. Correlations between variables were assessed by using Spearman's rank correlation corrected for multiple testing. A *P*-value < 0.05 was considered to be statistically significant. The cut-off values for FSAP-inhibitor complexes in healthy volunteers were calculated by a method described by Rümke *et al. *[26].

## Results

### FSAP activation by apoptotic and necrotic cells

**Figure 1:**
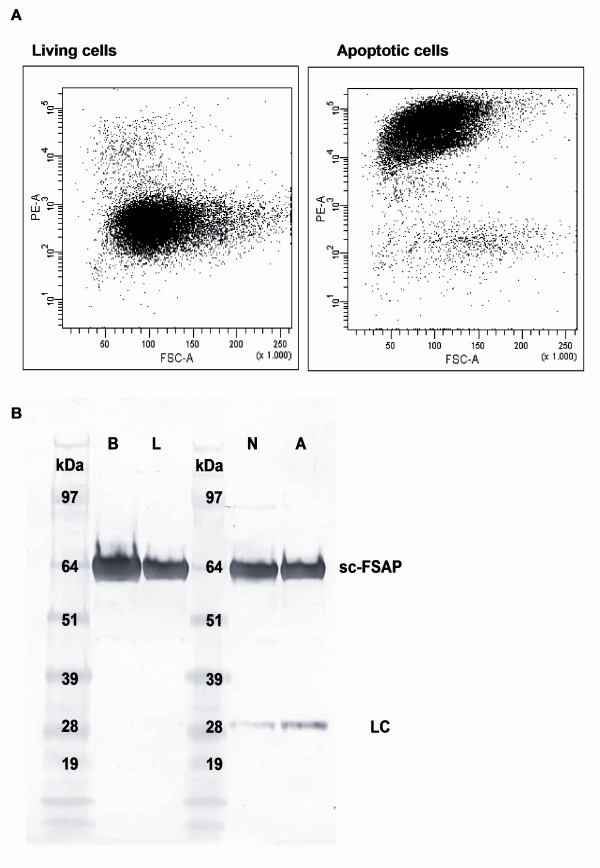
**FSAP binding and activation upon contact with apoptotic and necrotic cells**. After incubation of r-plasma with apoptotic and living cells, cells were incubated with an anti-FSAP antibody. Cells were stained with a PE-labeled rabbit-anti-mouse F(ab')2 antibody and FSAP binding was measured with flow cytometry (1A). Cells being double positive for propidium iodide and Annexin V were considered to be apoptotic (not shown). After incubation of r-plasma with apoptotic **(A)**, necrotic (N) and living (L) cells, FSAP was immunoprecipitated with anti-FSAP-4. R-plasma incubated with buffer **(B) **was used as a negative control. Samples were applied to SDS-PAGE 4 to 12% gel under reduced conditions and blotted onto a nitrocellulose membrane. Detection was performed by using biotinylated anti-FSAP-9 recognizing the light chain (1B). scFSAP, single chain FSAP; LC, light chain.

Since incubation of plasma with apoptotic cells induces nucleosome release via the serine protease activity of FSAP we argued that FSAP must become activated by interaction with those apoptotic cells [10]. We, therefore, analyzed whether FSAP binds to apoptotic cells. Indeed, plasma FSAP binds strongly to apoptotic cells whereas binding to living cells is negligible (Figure 1A). In addition, plasma FSAP also strongly binds to necrotic cells (data not shown). Furthermore, we could show that interaction of r-plasma with dead cells leads to conversion of single chain FSAP into active two-chain FSAP as indicated by the appearance of the light chain at 28 kDa (Figure 1B). No activation could be found upon incubation with living cells.

### Identification of plasma inhibitors of FSAP

Since serpins form covalent complexes with their target proteases, activated FSAP will presumably form covalent complexes with its target inhibitors in plasma [27]. Hence, measurement of these complexes would offer an easy and sensitive way to monitor FSAP activation in r-plasma or plasma. To identify which serpins form complexes with activated FSAP, r-plasma was incubated with apoptotic cells and applied to an anti-FSAP affinity column. Analysis of the eluate of the r-plasma activated with apoptotic cells by SDS-PAGE under non-reducing conditions demonstrated an additional band at 97 kDa as compared to the r-plasma that was not incubated with apoptotic cells (data not shown). MALDI-TOF peptide mass fingerprinting and MALDI-TOF/TOF peptide sequencing analysis of the 97 kDa band resulted in a positive identification for FSAP and α_2_-antiplasmin (AP) which is described to be an inhibitor of FSAP in purified systems [17]. Although C1-inh was reported to inhibit activated FSAP in plasma we were not able to show C1-inh to form complexes with FSAP on SDS-PAGE [16].

### ELISA to measure FSAP-inhibitor complexes as a measure of FSAP activation in plasma

**Figure 2:**
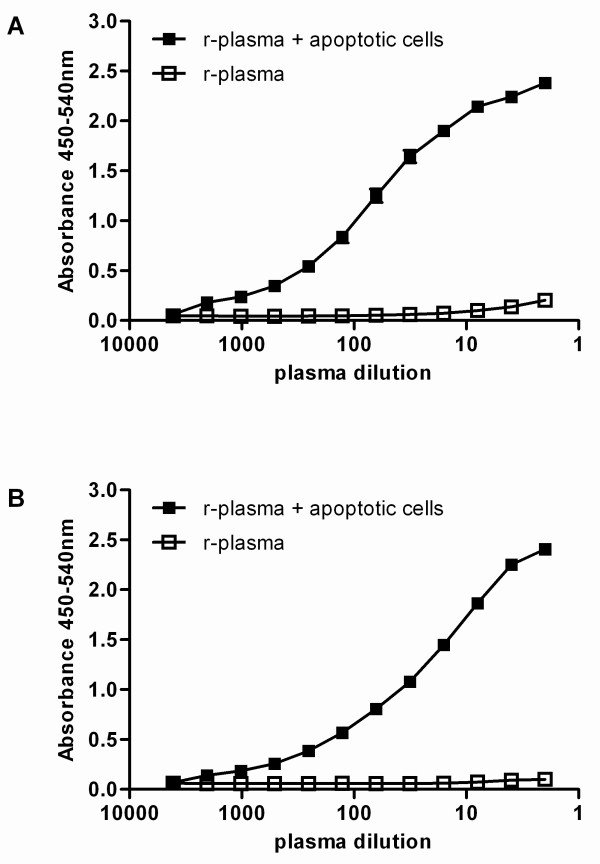
**ELISA's to measure FSAP complexes with C1-inhibitor and α_2_-antiplasmin**. To measure complexes of FSAP with AP, AAP-20 recognizing AP was used as catching antibody and biotinylated anti-FSAP-4 was used for detection **(A)**. To measure complexes of FSAP with C1-inh, KOK12, a monoclonal antibody recognizing complexed C1-inh was used as a catching antibody. As detection biotinylated anti-FSAP-4 was used **(B)**. Complexes were measured in r-plasma and r-plasma incubated with apoptotic cells. Results are given as mean ± SEM, (*n *= 3).

Our results demonstrate that upon activation with apoptotic cells FSAP forms covalent complexes with AP. To quantify these complexes we set up an ELISA to measure complexes between activated FSAP and AP. A monoclonal antibody recognizing AP was used as a catching and biotinylated anti-FSAP as detection antibody. Upon activation of FSAP in r-plasma, FSAP-AP complexes could be measured by ELISA, whereas no FSAP-AP complexes could be detected in plasma incubated with buffer (Figure 2A). No signal was detected when using an irrelevant antibody (anti-IL6) as detecting antibody demonstrating the ELISA to specifically detect FSAP-AP complexes (data not shown).

Since inhibition of FSAP with C1-inh was described by others [16], we also tested whether complexes between activated FSAP and C1-inh could be detected by ELISA. As a catching antibody a monoclonal antibody was used which exclusively reacts with C1-inh when complexed with its target proteases [24] and biotinylated anti-FSAP was used for detection. FSAP-C1-inh complexes could be measured in r-plasma after incubation with apoptotic cells whereas no FSAP-C1-inh complexes could be measured in r-plasma incubated with buffer (Figure 2B). Again no signal could be detected when an irrelevant antibody was used as detecting antibody.

These assays were used to monitor *in vitro *activation of plasma FSAP by cells. Citrated plasma of 20 healthy donors was incubated with apoptotic cells and FSAP-inhibitor complexes were measured. The median of the level of complexes in these controls was arbitrarily set as 0.5 AU/ml (FSAP-AP 0.5 ± 0.03, FSAP-C1-inh 0.5 ± 0.04).

**Figure 3:**
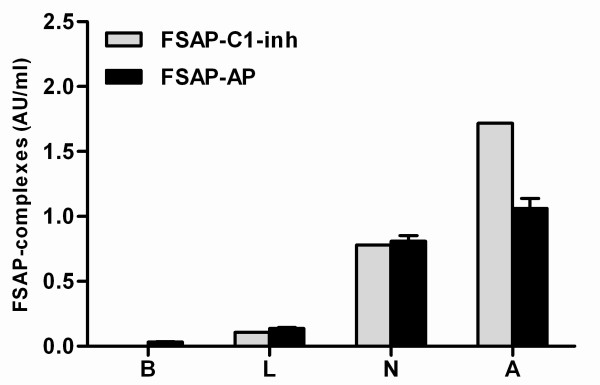
**FSAP complexes with C1-inhibitor and α_2_-antiplasmin as a measure of FSAP activation**. Living (L), necrotic (N) and apoptotic **(A) **cells were incubated with r-plasma for 30 minutes at 37°C. Thereafter complexes of FSAP with either AP or C1-inh were measured by ELISA. R-plasma incubated with buffer without cells **(B) **was used as a negative control. Results were expressed in AU/ml. Plasma of 20 healthy donors was incubated with apoptotic cells for 30 minutes at 37°C. Complexes of FSAP with either AP or C1-inh were measured by ELISA. The median of the level of FSAP-inhibitor complexes in these controls was arbitrarily set as 0.5 AU/ml and used as a reference. Results are given as mean ± SEM, (*n *= 3).

After incubation with apoptotic and necrotic cells complexes with C1-inh and with AP were readily detectable (Figure 3). Only low levels of FSAP-inhibitor complexes could be detected after incubation with living cells. No complexes could be detected after incubation of plasma with buffer. Together these results suggest that measurement of complexes of FSAP with its serpins, AP and C1-inh, by ELISA is a sensitive tool to assess FSAP activation in plasma.

### FSAP activation upon inflammation

Because FSAP is known to undergo autoactivation we extensively analyzed how plasma levels of FSAP complexes are influenced by sample preparation. Whereas purified plasma-derived FSAP is susceptible for autoactivation, FSAP in plasma turned out to be very resistant to autoactivation. Even blood clotting does not activate FSAP (data not shown). Moreover, plasma of sepsis patients stored for three hours at room temperature contains equal amounts of FSAP-serpin complexes as plasma immediately after collection (data not shown). When plasma samples of healthy donors are incubated for three hours at 37°C also no complexes are found. Only when plasma samples of patients were incubated at 37°C we found an increase in complex levels. Together these results indicate that there is no additional complex formation after collection at room temperature, nor via auto-activation, nor via circulating cell fragments in plasma of septic patients. We then measured FSAP activation in citrated plasma of patients with increasing severity of inflammation.

#### Patients with postoperative acute-phase response

**Figure 4:**
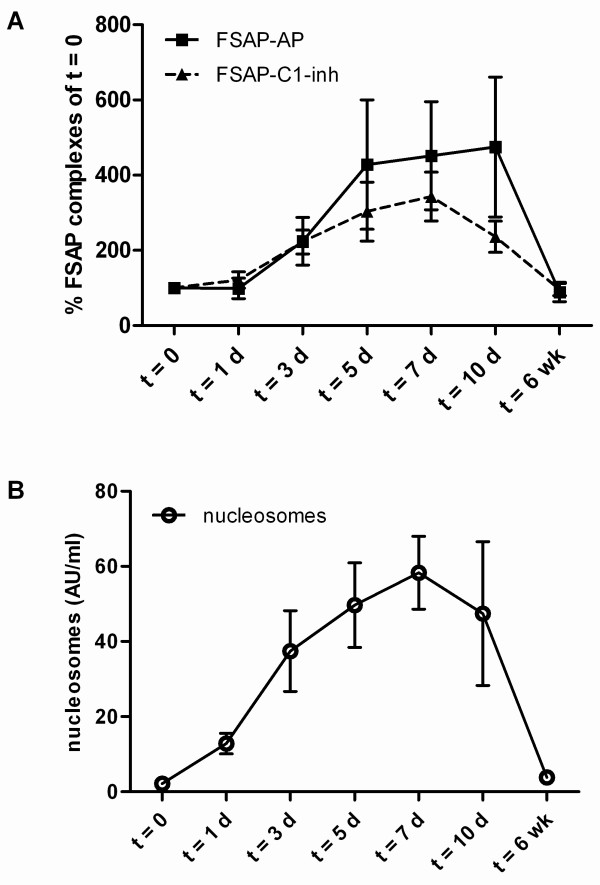
**FSAP-inhibitor complexes and nucleosomes in plasma of patients undergoing a transhiatal esophagectomy**. FSAP-AP and FSAP-C1-inh complexes and nucleosomes were measured in plasma from 20 patients undergoing esophageal resection with proximal gastrectomy, performed by a transhiatal approach without thoracotomy. Blood was sampled preoperatively (Day 0) and on days 1, 3, 5, 7, 10 after surgery and six weeks postoperatively. FSAP-inhibitor complexes were expressed as percentage of the amount FSAP-inhibitor complexes present at day 0. The absolute values at Day 0 were 0.51 AU/ml (range 0.03 to 4.46) for FSAP-AP and 0.06 AU/ml (0.01 to 0.40) for FSAP-C1-inh complexes. Nucleosomes were expressed as AU/ml. Results are given as mean ± SEM.

All 20 patients undergoing transhiatal esophagectomy were of Caucasian descent and 90% were males. The median age was 64.6 years (range 44.6 to 78.2 yrs). Postoperative levels of FSAP-AP and FSAP-C1-inh complexes increased from Day 0 with highest levels of FSAP-AP on Day 10 and of FSAP-C1-inh on Day 7 (Figure 4). FSAP activation correlated with the increase in nucleosome levels. The nucleosome levels correlated significantly with FSAP-AP complexes (r = 0.5539; *P *< 0.0001) and with FSAP-C1-inh complexes (r = 0.6440; *P *< 0.0001). We also measured FSAP antigen levels in these patients. It turned out that most patients showed a drop in FSAP levels on Day 1, returning to normal on Day 2. This drop is most likely not caused by consumption of FSAP but by the fluid balance in these patients. Serum IgM levels showed a similar drop on Day 1 (data not shown).

#### Patients suffering from severe sepsis and septic shock

**Figure 5:**
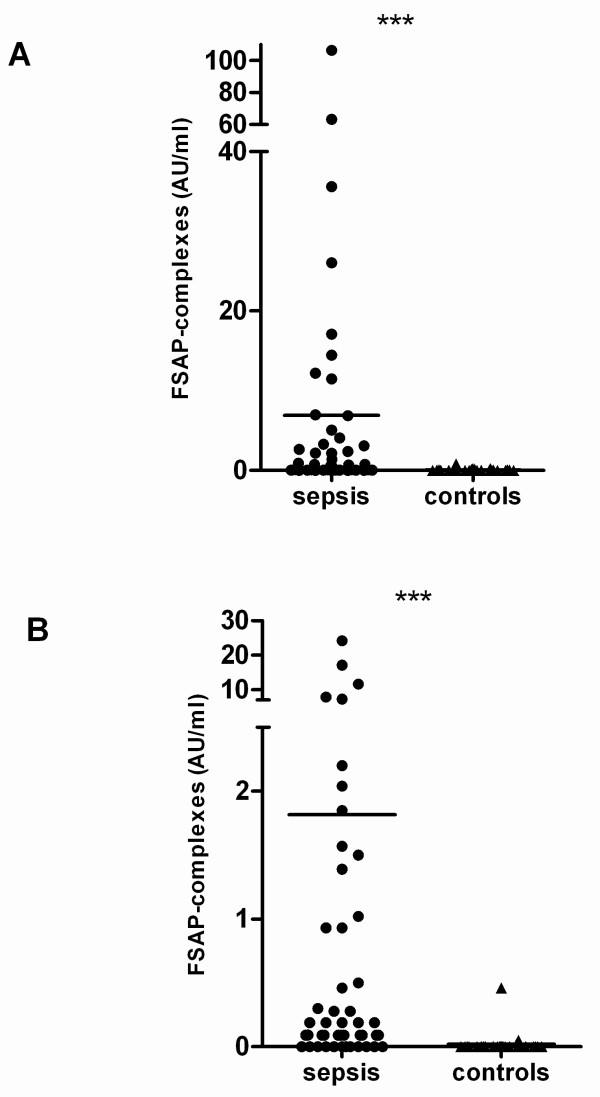
**FSAP-inhibitor complexes in plasma of sepsis patients and healthy controls**. FSAP-AP **(A) **and FSAP-C1-inh **(B) **complexes were measured in citrated plasma from 40 patients with severe sepsis or septic shock. FSAP-inhibitor complexes were expressed as AU/ml. Plasma of 20 healthy donors was taken as a control. Median values at a given time have been compared by using Mann Whitney Rank Sum test. *** indicates a *P *< 0.001.

Thirty-two patients with severe sepsis and 8 with septic shock were enrolled: 33 males (82.5%) and 7 females (17.5%) with a median age of 64.5 years (range 28 to 74 yrs). The clinical characteristics of these patients are extensively described elsewhere [22]. FSAP complexes with C1-inh and AP were elevated in 75% of the patients. The median complex levels were significantly higher in the sepsis patients as compared to the controls (Figure 5). There were no significant differences in the median levels of FSAP-inhibitor complexes between survivors (*n *= 27) and non-survivors (*n *= 13), nor for patients suffering from severe sepsis (*n *= 32) and septic shock (*n *= 8), respectively. FSAP antigen levels were lower in sepsis patients as compared to healthy controls (data not shown). Significant correlations between FSAP-inhibitor complexes as well as with nucleosomes could be found (Table 1). No or only weak correlation of FSAP-inhibitor complexes with clinical parameters, organ dysfunction scores and acute phase parameters, such as C-reactive protein or IL-6 could be found. In contrast, PAI-1 and C3a both being predictive parameters for outcome, significantly correlated with FSAP-inhibitor complexes (r > 0.33, *P *< 0.05).

**Table 1:** Correlations FSAP-inhibitor complexes and nucleosomes in patients suffering from severe sepsis and septic shock

	FSAP-AP		FSAP-C1-inh	
	
	r	*P*	r	*P*
**FSAP-AP**	-	-	0.905	< 0.0001
**FSAP-C1-inh**	0.905	< 0.0001	-	-
**Nucleosomes**	0.427	0.006	0.443	0.004

Correlations were calculated by using Spearman rank sum test and corrected for multiple testing. A *P*-value < 0.05 was considered to be statistically significant.

Abbreviations: AP, α_2_-antiplasmin; C1-inh, C1-inhibitor; FSAP: factor VII-activating protease.

#### Patients suffering from meningococcal sepsis

**Figure 6:**
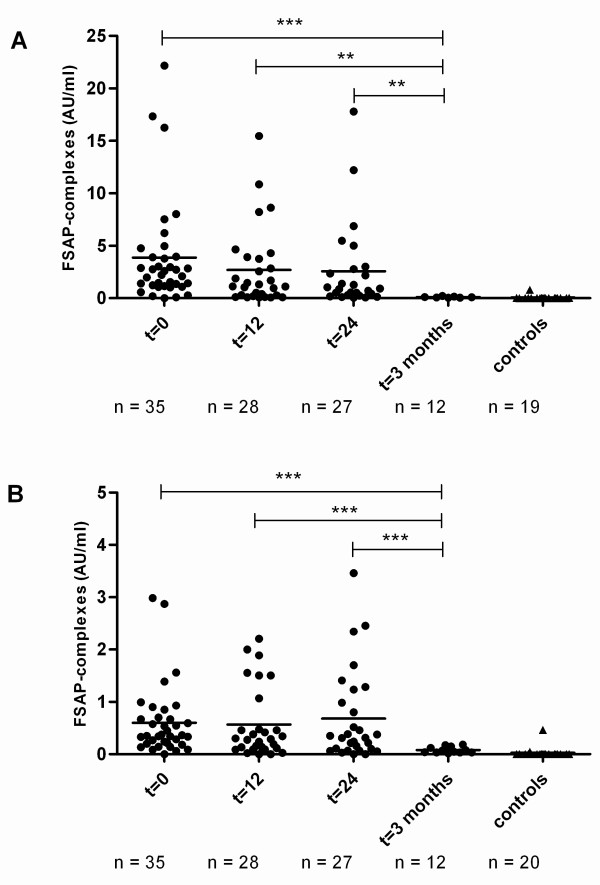
**FSAP-inhibitor complexes in plasma of children with meningococcal sepsis**. FSAP-AP **(A) **and FSAP-C1-inh **(B) **complexes were measured in citrated plasma from 38 meningococcal sepsis patients. Blood was sampled at 0 h, 12 h, 24 h and 3 months. FSAP-inhibitor complexes were expressed as AU/ml. Plasma of 20 healthy donors was taken as a control. Median values at a given time have been compared by using Mann Whitney Rank Sum test. *** indicates a *P *< 0.001, ** indicates a *P *< 0.01.

**Figure 7:**
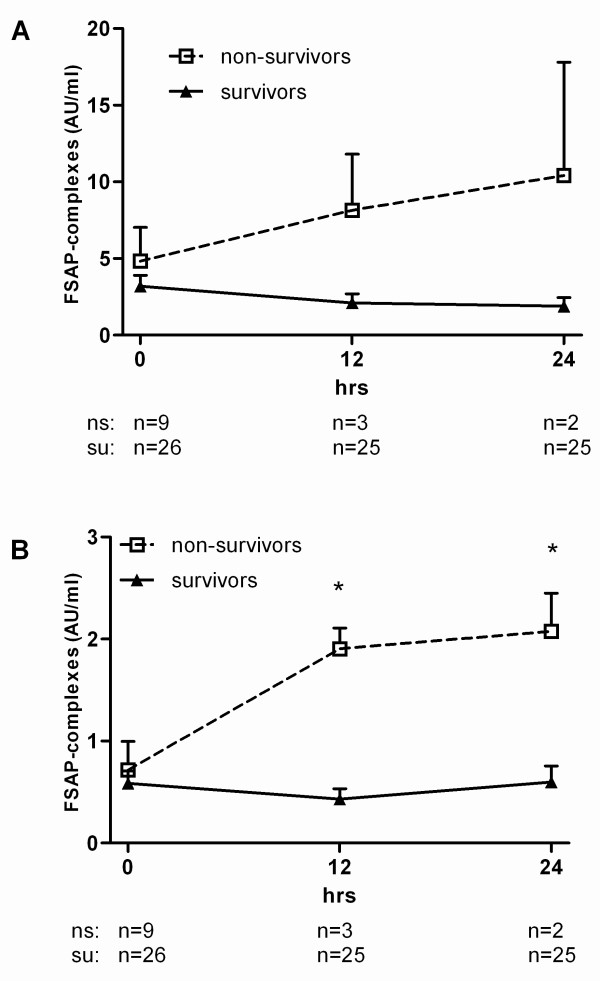
**FSAP-inhibitor complexes in plasma of survivors and non-survivors of meningococcal sepsis**. FSAP-AP **(A) **and FSAP-C1-inh **(B) **complexes were measured in citrated plasma from survivors and non-survivors of meningococcal sepsis. Blood was sampled at 0 h, 12 h and 24 h. FSAP-inhibitor complexes were expressed as AU/ml. The values are indicated by mean values ± SEM. Median values at a given time have been compared by using Mann Whitney Rank Sum test. * indicates *P *< 0.05.

Forty children with meningococcal sepsis were included in the study [23]. From these 40 patients, citrated plasma samples from 38 patients were available to measure FSAP-inhibitor complexes. From these 38 patients, 9 died as a result of the disease (non-survivors). The median age of the survivors (2.7 years (range 0.3 to 16.1 yr)) was higher (*P *= 0.021) than non-survivors (0.9 years (range 0.5 to 9.4 yr)). On admission, plasma samples were available from 35 patients. Increased FSAP complexes with C1-inh and AP were found in all patients at t = 0 (Figure 6). Significant differences were found in FSAP-AP and FSAP-C1-inh complex levels between t = 0, t = 12 h and t = 24 h compared to the levels at t = 3 months and in healthy controls. Furthermore significant correlations between FSAP-inhibitor complexes as well as with nucleosomes could be found (Table 2). FSAP-inhibitor complexes increased with fatality (Figure 7). FSAP-C1-inh complexes were significantly higher after 12 and 24 hours in non-survivors as compared to survivors. FSAP-AP complexes were also higher in non-survivors as compared to survivors, although the difference was not statistically significant. FSAP antigen levels were lower in children suffering from meningococcal sepsis as compared to healthy controls (data not shown).

**Table 2:** Correlations FSAP-inhibitor complexes and nucleosomes in children suffering from meningococcal sepsis

	FSAP-AP		FSAP-C1-inh	
	
	r	*P*	r	*P*
**FSAP-AP**	-	-	0.521	0.001
**FSAP-C1-inh**	0.521	0.001	-	-
**Nucleosomes**	0.717	< 0.0001	0.616	< 0.0001

Correlations were calculated by using Spearman rank sum test and corrected for multiple testing. A *P*-value < 0.05 was considered to be statistically significant.

Abbreviations: AP, α_2_-antiplasmin; C1-inh, C1-inhibitor; FSAP: factor VII-activating protease.

## Discussion

We recently demonstrated FSAP to induce nucleosome release from apoptotic cells [10]. There is growing evidence that the content of dead cells can act as endogenous mediators of inflammation [8]. Therefore, we were interested in measuring FSAP activation in disease. Direct measurement of FSAP enzyme activity in plasma is extremely difficult due to the lack of specific substrates. Moreover, it is to be expected that the half-life of active enzyme is extremely short, due to the presence of high plasma levels of C1-inh and AP in the plasma. In the present paper we demonstrate that FSAP activation in plasma can be monitored by assays detecting complexes between FSAP and its target serpins AP and C1-inh. We used these assays to demonstrate FSAP activation in post-surgery patients, patients suffering from severe sepsis, septic shock and meningococcal sepsis.

In purified systems C1-inh and AP were reported to be the main inhibitors of activated FSAP [11,16,17]. In plasma only C1-inh was demonstrated to be an inhibitor of FSAP [16]. Serpins form covalent complexes with their target proteases [27,28]. Therefore, FSAP in plasma is expected to form covalent complexes with its inhibitors upon activation. We showed by affinity purification and mass spectrometry analysis, that FSAP in plasma forms a complex with AP upon activation with apoptotic cells. In order to set up a more simple and sensitive method we tried to measure FSAP-inhibitor complexes by ELISA. Indeed, we were able to detect FSAP-AP complexes in plasma which is incubated with apoptotic cells. Since no FSAP complexes with AP could be detected in plasma which is not activated with apoptotic cells or by using an irrelevant detection antibody the ELISA specifically detects complexes of FSAP with AP. Although we were not able to detect FSAP-C1-inh complexes after affinity purification by SDS-PAGE, these complexes could be detected by ELISA. This might indicate that ELISA is a more sensitive method than affinity purification and SDS-PAGE or that the FSAP-C1-inh complex is dissociated upon SDS-PAGE.

FSAP strongly binds to apoptotic as well as to necrotic cells and no binding to living cells is seen. Probably this binding leads to activation of FSAP as shown by Western blotting and complex formation. Living cells lead to some complex formation, which is very likely due to the presence of dead cells in cultured cells. Indeed Figure 1A shows that living jurkat cells contain a fraction of dead cells that bind FSAP. The fact that FSAP activation by living cells is not seen in Western blot reflects the much higher sensitivity of the complex ELISAs to detect FSAP activation. To which structure FSAP binds, and how FSAP activation is achieved, is not clear yet. RNA, and to a lesser extent also DNA, is reported to activate FSAP [13,15]. We routinely use RNase-treated cells to induce FSAP activation. RNase treatment improves the specificity of the Propidium Iodide staining used in the Nucleosome releasing factor assay [10]. No differences in FSAP activation could be found between RNase treated and untreated cells suggesting that FSAP activation by RNA in our system is rather unlikely. Our results further show that when in plasma or serum, FSAP is a robust stable molecule. Once purified it becomes very susceptible to auto-activation. For a molecule described to be involved in coagulation and fibrinolysis is seems odd that even total coagulation of blood does not lead to FSAP activation.

Our results indicate FSAP activation to be a useful tool to measure cell death in circulation. Of course some questions remain unanswered. How exactly is FSAP activated, how much FSAP remains associated with dead cells and how much is released in the circulation? Another question is what determines the inhibitor specificity. Do different types of activation lead to different inhibitor complexes and to what extent is inhibitor concentration important? Notwithstanding these questions we think that the newly developed complex assays could be useful in understanding inflammation such as sepsis. Cell death is a central event in the pathogenesis of sepsis and is reflected by circulating nucleosomes [5,6]. Indeed, FSAP activation could be detected in severe sepsis, septic shock and meningococcal sepsis and significantly correlated with nucleosome levels. Even in a model of "a low grade inflammation" FSAP activation could be detected and significantly correlated with nucleosome levels, whereas FSAP complexes with C1-inh or AP could not be detected in healthy controls. Altogether these results suggest FSAP activation to be a sensor for cell death in circulation.

FSAP activation increases with the severity of inflammation as shown by FSAP-inhibitor complexes, which correlate with the increase in disease severity with the lowest level in post surgery and higher levels in adult patients with sepsis and children suffering from meningococcal sepsis. FSAP-inhibitor complex levels in meningococcal sepsis were significantly higher in survivors than in non-survivors, although the non-survivor group was small. FSAP-inhibitor complex levels in adult sepsis increased with the severity of inflammation as evidenced by significant correlations with inflammatory markers (C3a, PAI-1) but did not discriminate for fatality. The discrepancy between adults and children might be explained by the fact that the sepsis patients form a heterogeneous group composed of patients from surgical as well as from medical ICU (*n *= 22 vs *n *= 18), whereas the children suffering from meningococcal sepsis form a considerably more homogenous population with a clear-cut onset of sepsis.

## Conclusions

In summary, we show that FSAP in plasma is activated upon contact with dead cells and this activation can be followed by measuring FSAP-AP and FSAP-C1-inh complexes in plasma by ELISA. We demonstrate FSAP activation in adults suffering from sepsis and children with meningococcal sepsis which increases with the severity of inflammation. Our results suggest that FSAP activation in sepsis might be involved in nucleosome release thereby contributing to lethality.

## Abbreviations

AP: α_2_-antiplasmin; ARDS: acute respiratory distress syndrome; AT-III: antithrombin III; BCIP: 5'-bromo-4'-chloro-3'-indolyl phosphate; C1-inh: C1-inhibitor; DTT: dithiothreitol; FCS: fetal calf serum; FSAP: Factor VII-activating protease; HABP2: hyaluronic acid binding protein 2; HPE: high performance ELISA; NBT: nitro blue tetrazolium; PAI-1: plasminogen activator inhibitor-1; PC: plasma-derived protein C; r-plasma: recalcified citrated plasma; TMB: (3,5,3',5')-tetramethylbenzidine.

## Competing interests

The authors declare that they have no competing interests.

## Authors' contributions

FS and IB performed all experiments. SZ and LA supervised this work. FS, JH, MAB, JWOT, WAW, TP, LA and SZ were involved in analyzing data and preparing the manuscript.
